# Hydatid cyst in the wrist

**DOI:** 10.1590/0037-8682-0733-2020

**Published:** 2021-03-08

**Authors:** Rojbin Ceylan Tekin, Emin Özkul, Recep Tekin

**Affiliations:** 1 Mardin State Hospital, Department of Radiology, Diyarbakir, Turkey.; 2 Dicle University, Faculty of Medicine, Department of Trauma and Orthopedic Surgery, Diyarbakir, Turkey.; 3 Dicle University, Faculty of Medicine, Department of Infectious Diseases and Clinical Microbiology, Diyarbakir, Turkey.

A 41-year-old female presented with a 3-year history of swelling in the right wrist associated with pain and numbness involving the fingers. Physical examination of her wrist revealed swelling and mildly painful movement with minimal limitation. The patient was living in a region endemic for hydatid disease. A radiological study revealed soft tissue swelling of the wrist with no bone erosion or calcification (Figure 1). Magnetic resonance imaging of the wrist showed multiple round, multivesicular daughter cysts occupying almost the entire volume of the mother cyst that was confined to the soft tissue at the extensor surface surrounding the tendons and muscles of the medial wrist, including the extensor carpi radialis, extensor digiti minimi, extensor digitorum, and extensor pollicis longus, with no infiltration into the bone or surrounding neurovascular structures (Figure 2). Following the irrigation of the cystic cavity with a hypertonic saline solution, the cystic mass was completely excised with its capsule (Figure 3). The multiple daughter cysts were filled with a muddy substance, typical of hydatid disease. Histopathological examination confirmed the diagnosis of hydatid disease. The patient received albendazole at 15 mg/kg/day (3 and 1 weeks of treatment and nontreatment, respectively) for 6 weeks to prevent recurrence. Pericystectomy combined with neoadjuvant therapy could potentially help reduce complications and recurrence in soft tissue hydatid cysts^1^ ^,^ ^2^. Early recognition of hydatid cyst cases is critical in preventing complications^3^.

**FIGURE 1:**
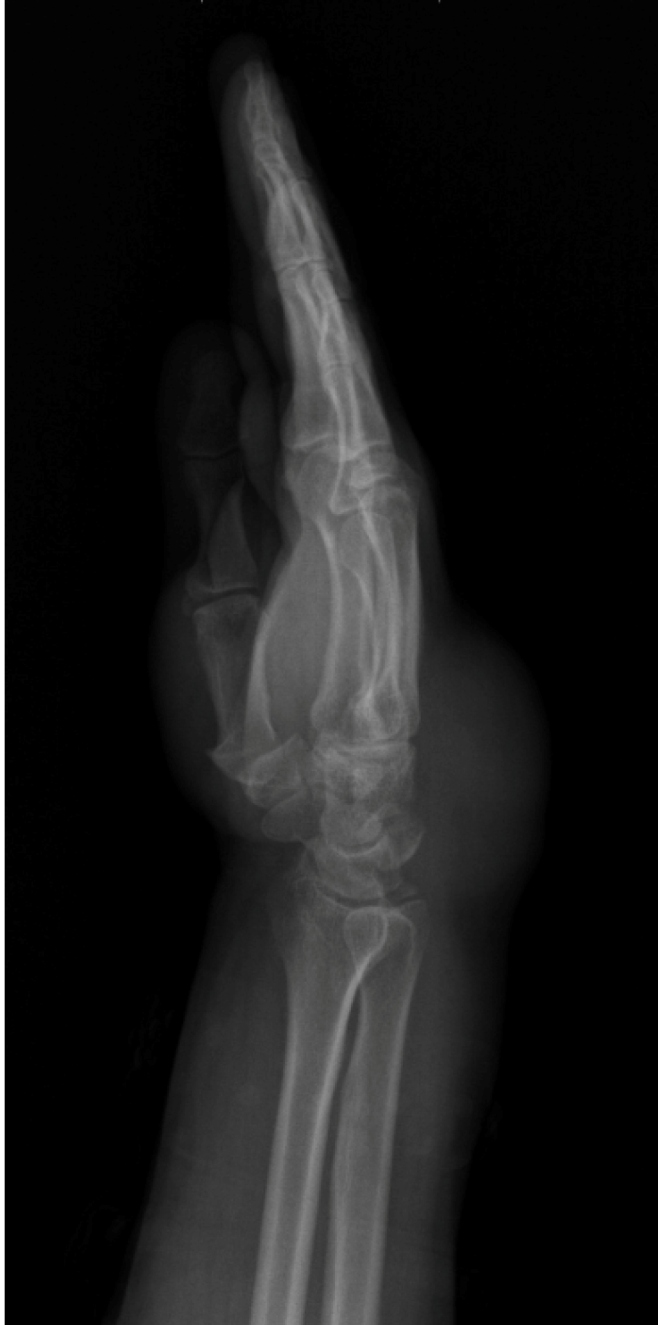
Radiological study revealing soft tissue swelling of the wrist.

**FIGURE 2:**
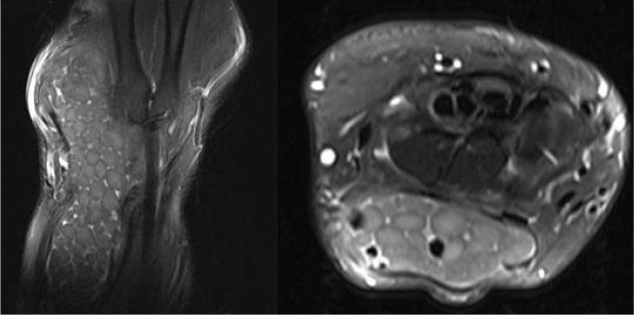
Magnetic resonance imaging of the wrist showing multiple, round, multivesicular daughter cysts occupying almost the entire volume of the mother cyst confined to the soft tissue at the extensor surface.

**FIGURE 3:**
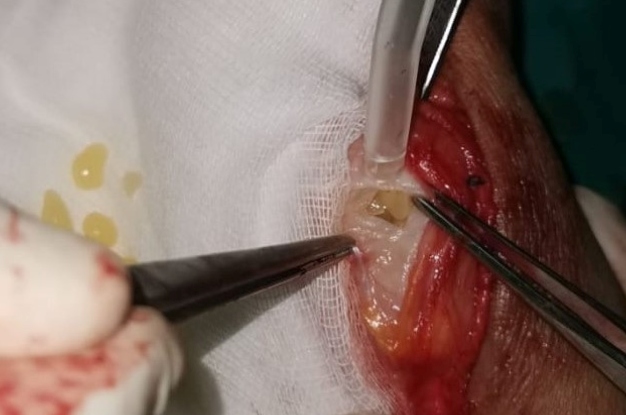
Multivesicular cyst containing multiple daughter cysts.

## References

[1] Tekin R, Onat S, Tekin RC (2016). Hydatid cysts in a patient with multiple organ involvement. Rev Soc Bras Med Trop.

[2] Tekin R, Avci A, Tekin Ceylan, Gem M, Cevik R (2015). Hydatid cysts in muscles: Clinical manifestations, diagnosis, and management of this atypical presentation. Rev Soc Bras Med Trop.

[3] Tekin R, Tekin RC, Avci A (2020). Giant hydatid cysts of the lung and liver. Rev Soc Bras Med Trop.

